# Correlation between the diameter of esophageal varices measured using a virtual ruler under endoscopy and portal pressure gradient

**DOI:** 10.3389/fmed.2024.1443581

**Published:** 2024-11-13

**Authors:** Yudi Mao, Zhongliang Fang, Yingying He, Jing Jin, Xiping Ding, Derun Kong

**Affiliations:** ^1^Department of Gastroenterology, The First Affiliated Hospital of Anhui Medical University, Anhui Province Key Laboratory of Digestive Diseases, Hefei, China; ^2^Department of Geriatrics and Gastroenterology, The First Affiliated Hospital of USTC, Division of Life Sciences and Medicine, University of Science and Technology of China, Hefei, Anhui, China; ^3^Continuous Education College, Anhui Medical University, Hefei, China

**Keywords:** portal pressure gradient, esophageal vein diameter, liver cirrhosis, esophageal varices, portal hypertension

## Abstract

**Background:**

Esophageal variceal diameter (EVD) is a crucial factor in determining the risk of esophageal variceal bleeding, which is associated with an increased portal pressure gradient (PPG). However, research into the relationship between EVD and PPG has been limited, primarily because the assessment of EVD depends on visual estimation during endoscopy. Recently, we developed an artificial intelligence (AI)-based method to accurately detect EVD. In this study, we aim to investigate the correlation between EVD and PPG, with the goal of evaluating EVD as a potential non-invasive indicator of PPG.

**Methods:**

This study included both retrospective and prospective data from 128 patients diagnosed with portal hypertension and gastroesophageal varices, gathered from two medical institutions. Clinical data including PPG, biochemical markers, and routine blood tests were collected. In the retrospective phase, EVD was evaluated using an AI-based virtual ruler. In the prospective phase, PPG was measured using radiological intervention methods, and EVD was measured during endoscopy with the aid of AI.

**Results:**

A positive correlation between PPG and EVD was identified (*r* = 0.521, *P* < 0.001), which was further supported by multivariate linear regression analysis (b = 6.521, t = 6.872, *P* < 0.001). When patients were stratified into two groups based on PPG levels (27 patients with PPG < 20 mmHg and 101 patients with PPG ≥ 20 mmHg), a significant difference in EVD was observed between the groups (OR = 29.275, 95% CI 5.590–153.304, *P* < 0.001), with larger EVD in the higher PPG group. These findings suggest that EVD may serve as a predictor of adverse events associated with elevated PPG levels. In addition, receiver operating characteristic (ROC) curve analysis showed that EVD had an accuracy of 0.814 in diagnosing PPG function (standard error 0.048, 95% CI 0.720-0.908; P < 0.001), indicating that PPG levels are likely to exceed 20 mmHg when the variceal diameter is greater than 1.1 cm.

**Conclusion:**

EVD demonstrated a positive correlation with PPG and could potentially be used as a predictive marker for assessing PPG levels. These findings provide novel insights for the non-invasive evaluation of PPG in clinical practice.

## Introduction

Portal hypertension (PH), a major pathological consequence of cirrhosis, leads to various clinical manifestations and severe complications, including ascites, varices, variceal bleeding, hepatic encephalopathy, and even cardiac and pulmonary complications. Bleeding from gastroesophageal varices is a common and life-threatening complication of liver cirrhosis, with the highest and most concerning mortality rates reported (1, 2). Due to the poor prognosis associated with variceal bleeding, previous studies and clinical guidelines have focused on identifying predictors and implementing preventive interventions for patients at high risk of bleeding. The primary goal of screening and monitoring is to identify individuals at high risk of esophageal variceal bleeding (EVB), thereby facilitating the timely implementation of prevention strategies.

Portal pressure gradient (PPG) and hepatic venous pressure gradient (HVPG) are recognized as the gold standard for diagnosing PH. The levels of PPG or HVPG are closely associated with the development of varices, variceal bleeding (3, 4), shunt dysfunction, and patient survival (5). However, measuring PPG or HVPG is invasive, time-consuming, and costly, making it impractical for routine clinical use. Patients may also be reluctant to undergo these tests solely for examination purposes. Therefore, the search for non-invasive tools to predict the development or bleeding of esophageal varices (EV) has garnered significant interest among clinical professionals. Researchers have discovered that non-invasive methods are increasingly preferred over PPG or HVPG in clinical practice. For instance, evidence has shown that the severity of varices with red wale marks is linked to an increased risk of bleeding (6), and liver stiffness combined with the spleen/platelet ratio is associated with varices requiring treatment (7).

It is well established that esophageal variceal pressure rises with increasing portal vein pressure (PVP). According to Laplace's law, the tension in the blood vessel wall is positively correlated with the square of the radius (r^2^), making vessel diameter a critical factor among several influencing variables. An increase in diameter results in thinning of the blood vessel wall and the appearance of red wale marks. When the tension exceeds a certain threshold, the vessel wall ruptures, resulting in bleeding. While current evidence suggests that an increase in esophageal variceal diameter (EVD) can predict the risk of bleeding, the correlation between EVD and HVPG or PPG has not yet been established. If the bleeding risk threshold of HVPG or PPG could be accurately predicted through EVD, it would significantly facilitate the prediction of bleeding and prognosis using endoscopic examination.

Despite significant updates in the management of gastroesophageal varices in recent years, particularly in the use of non-invasive methods to assess the degree of PH, it is essential to continuously identify factors that predict the risk of EVB and long-term prognosis. According to our previous research, EVD can be determined accurately using a non-invasive technology, called a virtual ruler (VR), supported by artificial intelligence (AI) developed by our team.

We also found that EVD significantly reduced rebleeding rates after endoscopic variceal ligation, and the risk of rebleeding increased notably when EVD exceeded 1.35 cm (8). Our study aimed to evaluate and establish the correlation between PPG and EVD through endoscopic examination. This involved multicenter retrospective and prospective analyses to predict PPG by measuring EVD endoscopically. The goal was to provide clinical evidence for predicting bleeding risk using non-invasive methods.

## Materials and methods

### Patients

The study encompassed data from both retrospective and prospective research, involving a total of 128 patients with PH and gastroesophageal varices. In the retrospective phase, 64 patients who underwent transjugular intrahepatic portosystemic shunt (TIPS) creation were selected from the First Affiliated Hospital of Anhui Medical University. This part of the study was conducted from July 2019 to January 2022. In the prospective phase, 12 patients who underwent TIPS creation were also selected from the same medical institution from March 2022 to January 2023, while 52 patients who received endoscopic selective varices devascularization were selected from the First Affiliated Hospital of the University of Science and Technology of China (USTC) from February 2022 to August 2023.

The clinical analysis was approved by the ethics committee of the First Affiliated Hospital of Anhui Medical University (NO. PJ20221016) and the Medical Research Ethics Committee of the First Affiliated Hospital of USTC (NO. PJ024E45). The inclusion criteria were as follows: (1) age between 18 and 75 years, (2) confirmation of EV through endoscopy, and (3) normal diameter of the hepatic vein and inferior vena cava. The exclusion criteria were as follows: (1) acute infection status, (2) use of medications affecting PVP in the past week, and (3) presence of portal vein thrombosis.

### Clinical data collection

Patients' clinical data were collected from the Electronic Medical Record System or during the general treatment process, including etiology, spleen size, Child-Pugh score, prothrombin time (PT), serum levels of alanine aminotransferase (ALT), total bilirubin (TBiL), albumin (ALB), creatinine (Cr), white blood cell (WBC) count, hemoglobin (HB), platelet (PLT) count, and the presence of ascites. Ascites were categorized into four levels based on the depth of fluid observed via abdominal ultrasound: no ascites (level 0, none), small amounts of ascites (level 1, 0–30 mm), moderate amounts of ascites (level 2, 30–60 mm), and large amounts of ascites (level 3, more than 60 mm). The Child-Pugh grade is represented as a numerical value in statistical analysis: Child-Pugh A as grade 1, Child-Pugh B as grade 2, and Child-Pugh C as grade 3.

### Measurement of PPG

For the retrospective data, the information regarding PPG was extracted from the Electronic Medical Record System. In the prospective phase, PPG was measured by attending interventional radiologists with more than 10 years of experience or senior physicians. The procedures were performed after clarifying the patient's diagnosis and clinical condition. For patients undergoing endoscopic selective varices devascularization, the left branch of the portal vein was punctured under ultrasound guidance to inject an appropriate contrast agent for portal vein imaging.

An introducer sheath and microwires were inserted into the portal vein to measure PVP. The right internal jugular vein approach was then used with a balloon catheter to measure wedged hepatic venous pressure (WHVP). After releasing the balloon, free hepatic vein pressure (FHVP) and inferior vena cava pressure (IVCP) were measured. The PPG was calculated using the formula: PPG = PVP − IVCP. Preoperative and postoperative PVP and IVCP were measured using the transjugular approach before performing venous shunt surgery for patients undergoing TIPS. The pressure unit conversion formula is 1 mmHg = 0.735 cmH_2_O.

### Measurement of EVD

Endoscopic examinations were conducted in all cases, mostly within 1 week before or after the PPG measurements and never exceeding 1 month. A non-invasive technology called VR, supported by AI, was used to measure the diameter of esophageal varices during endoscopy. This technological innovation was developed by our team in a previous study (9). A transparent cap (cat. no. DL-108-40; Micro Tech Co. Ltd.) with an inner diameter of 1 cm was affixed to the tip of the endoscope (cat. no.GIF Q260J; Olympus), and the VR was activated during the endoscopic examination.

**Figure 1:**
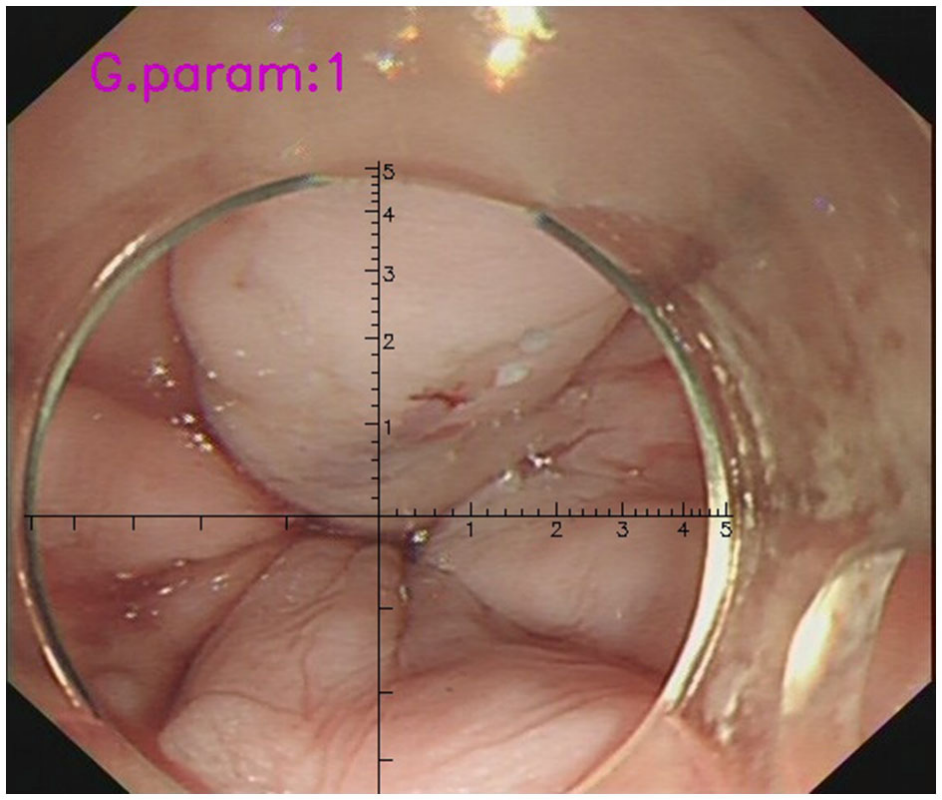
The measurement of EVD during gastroscopy with the assistance of VR. AI-assisted software could detect the discontinuous arc of the cap and establish a coordinate system at the center of the circle automatically (the cursor has a scale of 1mm per scale). The diameter of the target EV in the picture was determined to be approximately 0.6 cm.

The cursor continuously adjusted with the movement of the endoscope, aligning with the transparent cap's discontinuous arc and automatically establishing a coordinate system at the center of the circle (Figure 1). Testing demonstrated that the software package proved to be a valuable and dependable tool for the endoscopic identification and treatment of EV in patients with liver cirrhosis, leading to the acquisition of the National Utility Model Patent (NO. CN115345850A, CN115311239A, CN115345851A). As some patients presented with both small and large EVs, the large varices were selected for analysis. EVD was retrospectively reexamined using a VR or measured during gastroscopy procedures using AI-assisted software, which was jointly operated and averaged by two endoscopists, one of intermediate and the other of senior level, who had undergone the relevant training on the VR technology.

### Statistical analysis

This study utilized Statistical Program for Social Sciences (SPSS) 20.0 software (IBM, SPSS, Inc., Chicago, IL, USA) for statistical analysis. The measurement data followed a normal distribution and were presented as mean ± standard deviation, M ± SD. For non-normal distribution or ordinal data, the median and interquartile range were used M (P25, P75). An independent samples t-test was used to compare rates and means for normally distributed data, while a non-parametric test was applied for skewed distributions and categorical data. Spearman correlation analysis and binary logistic regression models were utilized to examine the relationship between the diameter of EVD and PPG. The receiver operating characteristic (ROC) curve analysis was performed to assess the accuracy of the esophageal variceal diameter to predict PPG and to determine the cutoff values based on sensitivity and specificity. Statistical significance was determined at a P-value of < 0.05.

## Results

### Case information

The data were collected from a total of 128 patients across two medical institutions, adhering to strict inclusion criteria. This cohort comprised 35 women and 93 men. The primary cause of cirrhosis and PH was hepatitis B virus, accounting for the majority of cases (85, 66.4%), followed by alcoholic cirrhosis (7, 5.5%), autoimmune hepatitis (4, 3.1%), hepatitis C virus (4, 3.1%), and some other causes, such as drug-induced cirrhosis and hepatolenticular degeneration. Additionally, patients with unexplained liver cirrhosis contributed to a significant proportion (23, 18.0%). More detailed patient characteristics are presented in Tables 1, 2.

**Table 1:** Information on all cases.

**Cases**	**Shapiro-Wilk Test**	***χ ±s***	***M* (*P_25_, P_75_*)**
Age (year)	0.200	55.02 ± 11.23	-
PPG (mmHg)	0.223	24.34 ± 4.88	-
SBP (mmHg)	0.200	105.12 ± 9.53	-
DBP (mmHg)	0.058	62.80 ± 7.54	-
EVD (cm)	0.002	-	1.20 (0.80, 1.50)
ALB (g/L)	0.944	31.57 ± 5.96	-
Ascites (grade)	0.000	-	1 (1, 2)
ALT (U/L)	0.000	-	25.00 (17.00, 41.00)
BA (umol/L)	0.005	-	47.75 (25.00, 65.65)
PT (s)	0.016	-	15.20 (13.70, 17.40)
WBC (× 10^9^/L)	0.000	-	2.41 (1.56, 3.61)
HB (g/L)	0.033	-	73.00 (63.00, 84.00)
PLT (× 10^12^/L)	0.000	-	60.00 (38.00, 83.00)
TBiL (umol/L)	0.000	-	20.11 (14.93, 28.98)
Child-Pugh score	0.000	-	8.00 (7.00, 9.88)
Child-Pugh grade	0.000	-	2 (2, 2)

PPG, portal pressure gradient; SBP, systolic blood pressure; DBP, diastolic blood pressure; EVD, the diameter of esophageal varices; ALB, albumin; ALT, alanine aminotransferase; BA, blood ammonia; PT, prothrombin time; WBC, white blood cell; HB, hemoglobin; PLT, platelet; TBiL, total bilirubin.

**Table 2:** Baseline comparison of clinical data from different sources.

**Factors**	**Research type**				**Medical institution**			
	**Retrospective (*****n*** = **64)**	**Prospective (*****n*** = **64)**	***t*** **/** ***Z***	***P***	**Institution1 (*****n*** = **52)**	**Institution2 (*****n*** = **76)**	***t*** **/** ***Z***	***P***
Age	53.16 ± 10.39	56.89 ± 11.81	1.900	0.060	56.98 ± 11.81	53.68 ± 10.69	1.642	0.103
Gender	1 (1, 1)	1 (1, 2)	−1.383	0.167	1 (1, 2)	1 (1, 1.75)	−0.716	0.474
SBP	104.80 ± 9.88	105.44 ± 9.22	0.379	0.705	105.58 ± 9.44	104.80 ± 9.63	0.450	0.653
DBP	62.47 ± 7.21	63.14 ± 7.90	0.502	0.616	63.90 ± 7.91	62.04 ± 7.23	1.392	0.166
EVD	1.20 (0.80, 1.30)	1.20 (0.85, 1.50)	−1.774	0.076	1.20 (0.80, 1.58)	1.20 (0.85, 1.38)	−1.423	0.155
PPG	24.36 ± 4.57	24.32 ± 5.21	−0.046	0.964	23.51 ± 5.18	24.91 ± 4.62	−1.603	0.111
AST	23.00 (17.00, 40.75)	25.50 (19.25, 45.00)	−1.228	0.219	27.00 (21.00, 45.00)	23.00 (16.25, 45.75)	−1.452	0.147
BA	43.50 (17.75, 63.25)	52.25 (36.25, 92.50)	−1.201	0.230	47.70 (35.98, 68.25)	49.00 (18.00, 67.00)	−0.214	0.830
PT	16.2 (14.10, 18.50)	14.65 (13.40, 16.28)	−2.780	0.005	14.65 (13.33, 16.10)	16.00 (13.90, 18.30)	−2.581	0.001
WBC	2.31 (1.66, 3.33)	2.44 (1.51, 3.49)	−0.593	0.553	2.51 (1.12, 3.43)	2.28 (1.53, 3.71)	−0.338	0.735
PLT	65.00 (37.75, 89.00)	55.50 (39.00, 81.25)	−0.798	0.425	60.50 (44.25, 83.00)	58.50 (34.00, 84.00)	−0.756	0.450
TBiL	20.58 (13.03, 30.90)	19.55 (15.75, 27.10)	−0.312	0.755	19.40 (15.03, 27.10)	20.58 (13.12, 30.90)	−0010	0.992
ALB	30.33 ± 5.64	32.81 ± 6.06	2.396	0.018	32.46 ± 5.87	30.97 ± 5.99	1.392	0.161
Ascites	2 (1, 3)	1 (1, 2)	−2.143	0.032	2 (1, 3)	1 (1, 2)	−2.217	0.027
HB	68.50 (62.50, 84.00)	75.00 (62.00, 85.25)	−1.575	0.115	75.00 (63.00, 90.75 )	71.00 (61.00, 84.00)	−1.101	0.271
Child–Pugh score	8.00 (7, 10)	8.25 (7, 9)	−0.771	0.441	9.00 (7.00, 9.88)	8.00 (7.00, 9.75)	−0.061	0.951
Child–Pugh grade	2 (2, 3)	2 (2, 2)	−1.378	0.168	2 (2, 2.75)	2 (2, 2)	−0.223	0.823

### The correlation between PPG and EVD

**Figure 2:**
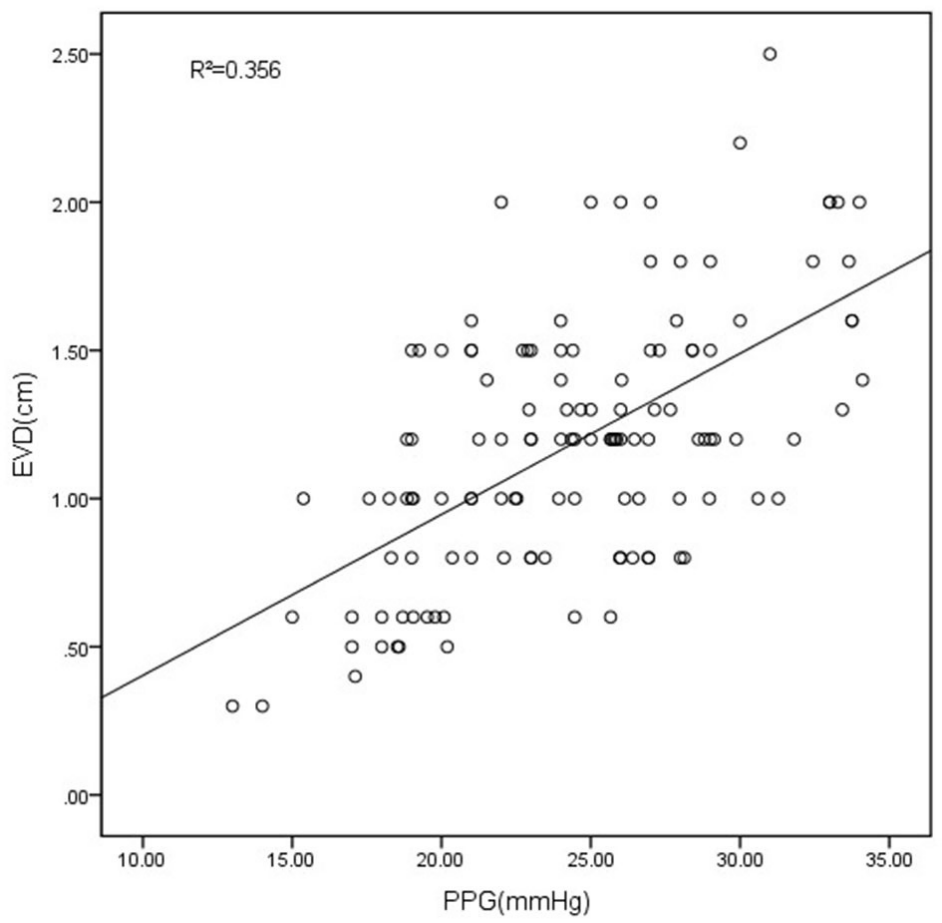
The scatter plot showed the correlation between PPG and EVD. EVD measured with VR had a good correlation with the PPG (R^2^ = 0.356, *P* < 0.001).

The measurement of diameters using the VR showed significant agreement between two endoscopists (r = 0.994, *P* < 0.001). The Spearman correlation analysis was performed to evaluate the correlation between EVD and PPG. The results indicated a positive correlation between PPG and EVD (correlation coefficient r = 0.521, *P* < 0.001), suggesting that EVD gradually increases with rising PPG. Furthermore, a scatter plot was utilized to illustrate the relationship between the two variables (Figure 2).

To minimize the influence of confounding factors on the research outcomes, variables such as PT, ALT, ALB, TBiL, WBC count, and ascites were incorporated into the construction of a multivariate linear regression model. The results also demonstrated a positive correlation between EVD and PPG (b = 6.521, t = 6.872, *P* < 0.001).

### Predicting PPG through EVD

Previous studies have indicated that as PPG increases, the risk of EVB gradually rises, with PPG ≥ 20 mmHg recognized as a criterion for poor prognosis (10). In the current study, clinical data were classified into two groups based on the aforementioned criteria, with 27 cases in the PPG < 20 mmHg group and 101 cases in the PPG ≥ 20 mmHg group. T-tests, or non-parametric tests, were employed to analyze the differences between the two groups (Table 3). The results revealed significant variances in EVD (*P* < 0.001), ascites (*P* = 0.036), and white blood cell count (*P* = 0.027).

**Table 3:** General material analysis of the two groups of patients.

**Factors**	**PPG < 20 mmHg (*n* = 27)**	**PPG ≥20 mmHg (*n* = 101)**	***t*/*Z***	***P***
Age	55.78 ± 11.09	54.82 ± 11.32	0.392	0.696
SBP	104.67 ± 11.10	105.24 ± 9.12	−1.831	0.075
DBP	60.33 ± 8.04	63.47 ± 7.31	−1.937	0.055
EVD	0.60 (0.50, 1.00)	1.20 (1.00, 1.50)	−5.038	0.000
ALB	29.93 ± 6.94	32.01 ± 5.63	−1.624	0.107
Ascites	1 (0, 2)	2 (1, 2)	−2.096	0.036
ALT	23.00 (17.00, 39.00)	25.00 (17.00, 47.50)	−0.678	0.498
BA	44.35 (36.00, 63.75)	49.50 (23.40, 71.95)	−0.296	0.767
PT	16.25 (13.55, 17.62)	15.10 (13.70, 17.20)	−1.069	0.285
WBC	3.09 (2.04, 5.17)	2.31 (1.51, 3.33)	−2.208	0.027
HB	69.00 (61.75, 77.75)	74.00 (63.00, 86.50)	−0.762	0.446
PLT	71.50 (39.75, 89.75)	58.00 (37.25, 74.75)	−1.468	0.142
TBiL	22.80 (16.40, 37.17)	18.80 (14.20, 27.00)	−1.486	0.137
Child-Pugh score	9 (7, 10)	8 (7, 9)	−0.560	0.576
Child-Pugh grade	2 (2, 3)	2 (2, 2)	−0.578	0.564

In our study, a binary logistic regression model was utilized to control for confounding factors on the outcomes. With PPG as the dependent variable, EVD, ALB, ascites, ALT, blood ammonia, PT, WBC, PLT, and TBiL were included to formulate a multivariate logistic regression equation. The results showed that EVD had a statistically significant difference between the two groups (OR = 29.275, 95% CI 5.590–153.304, P < 0.001), with a wider EVD observed in the higher PPG group. These results suggest that EVD can predict adverse events linked to elevated PPG levels, with the risk of bleeding increasing 3.377 times for every 1 mm increase in EVD. Detailed logistic regression analysis results are outlined in Table 4.

**Table 4:** Logistic regression analysis.

**Factors**	***P*-value**	***SE***	***Waldχ* ^2^**	***P***	***OR***	**95%*CI***
EVD	3.377	0.845	15.978	0.000	29.275	5.590–153.304
ALB	0.038	0.066	0.321	0.571	1.038	0.912–1.182
Ascites	0.372	0.340	1.197	0.074	1.450	0.745–2.823
SBP	−0.013	0.039	0.107	0.744	0.987	0.914–1.066
DBP	0.070	0.057	1.498	0.221	1.073	0.959–1.200
WBC	−0.121	0.142	0.727	0.394	0.886	0.670–1.171
Child-Pugh grade	0.394	0.724	0.297	0.586	1.483	0.359–6.128
TBiL	−0.030	0.021	1.920	0.166	0.971	0.931–1.012

**Figure 3:**
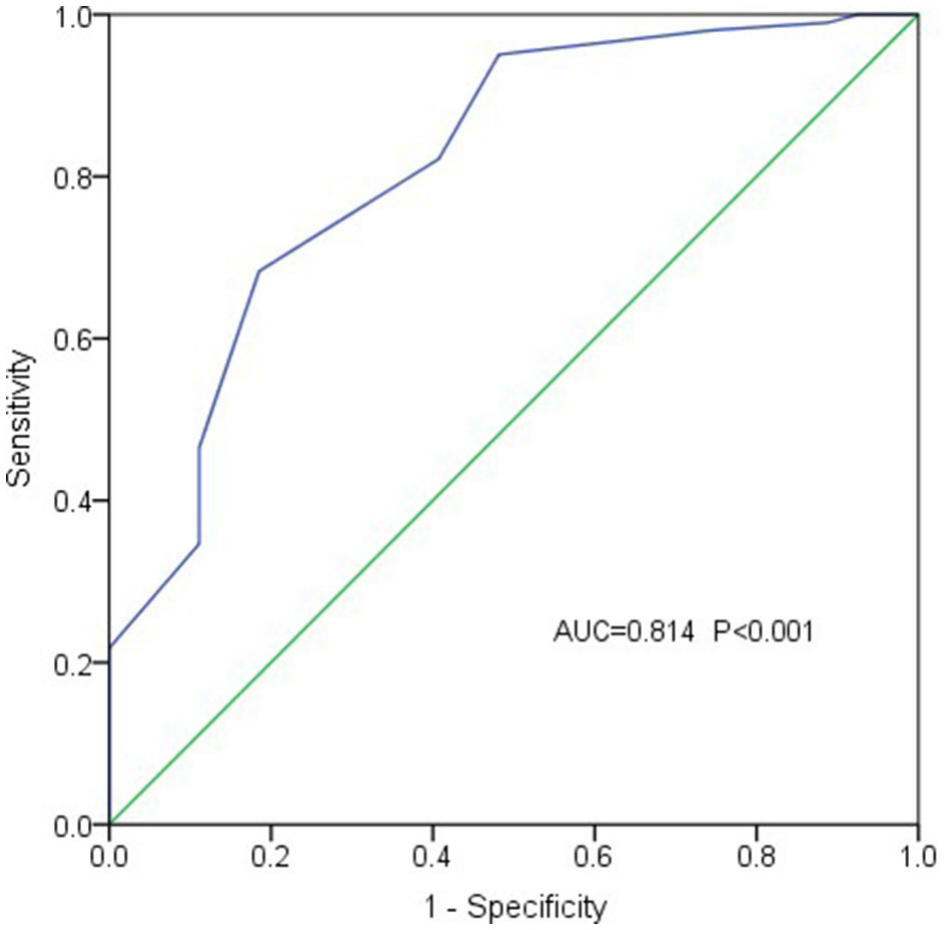
The ROC curve demonstrated that the EVD was effective in diagnosing PPG (PPG ≥ 20 mmHg).

The ROC curve analysis (with EVD as the test variable and PPG exceeding 20 mmHg as the state variable) demonstrated that EVD had an accuracy of 0.814 in diagnosing PPG function (standard error 0.048, 95% CI 0.720–0.908; *P* < 0.001) (Figure 3). An EVD cutoff value of 1.1 cm provided 68.3% sensitivity and 81.5% specificity in diagnosing PPG ≥ 20 mmHg, suggesting that PPG levels might exceed 20 mmHg when EVD exceeded 1.1 cm.

## Discussion

EVB is a common complication of liver cirrhosis and is closely related to the mortality and morbidity of these patients. The reported mortality rate from acute bleeding episodes accounts for ~15%−20% of patients with cirrhosis (11). Additionally, the endoscopic grade of EV closely correlates with EVB (12). A recent literature review found few reports on the relationship between PPG and EVD based on endoscopic findings. Therefore, in the present study, the correlation between PPG and EVD was determined through multivariate analysis.

PPG or HVPG plays an important role in the recognized standards for disease assessment and prognosis evaluation of PH. Variceal bleeding occurs at HVPG > 12 mmHg or with the appearance of other complications (13); an HVPG > 20 mmHg is a significant prognostic indicator of failure to control bleeding and mortality (10). Reports also suggest that HVPG is the only factor affecting the prognosis of EVB, and it has been demonstrated that there is a significant difference between the bleeding and non-bleeding groups using HVPG ≥ 20 mmHg as a cut-off indicator. In our study, the cases were divided into two groups based on the PPG, and the results showed that the level of EVD was positively correlated with the PPG. Furthermore, the multivariate analysis revealed a significant difference between the two groups. Therefore, this finding indicates that EVD is a predictive factor for adverse events, such as bleeding and poor prognosis, due to increased PPG, especially when EVD exceeds 1.1 cm.

In recent years, there have been significant advancements in the management of EVB, particularly in regard to non-invasive methods to assess the degree of PH, with a focus on the condition of varices. The most direct approach to evaluating EV is through endoscopic examination. The American Association for the Study of Liver Disease recommend endoscopic screening for patients with liver cirrhosis to identify those at a high risk of variceal bleeding (14). Research conducted over a decade ago revealed a correlation between HVPG and the severity of liver disease and the size of varices, demonstrating a significant difference in HVPG between small and large EVs (15). Subsequent studies also confirmed a strong positive correlation between the endoscopic grade of EV and HVPG (16). Another study indicated that EVD could predict early postoperative rebleeding in patients undergoing endoscopic variceal ligation, showing better predictive ability than the grade of EV. However, current reports have not established a relationship between EVD and PPG or HVPG, nor the potential clinical significance of diameter. EVD is a measurable indicator that is easier to quantify compared to the endoscopic grade of EV. The novelty of this study is its first-time description of the relationship between variceal diameter and PPG.

This study offers new insights into non-invasive techniques for measuring PPG, which could not only reduce patient discomfort and medical expenses but also assist clinical practitioners in assessing the severity of patient conditions.

The assessment of the diameter and severity of EV primarily depends on the visual judgment of doctors during endoscopic examinations. This method is subjective and lacks consistency, making it challenging to obtain accurate quantitative measurements. Variations in the diagnosis and grading of EV among different endoscopists have been observed (17).

To enhance data quality and improve the credibility of results, our team developed a non-invasive technology called the Virtual Ruler (VR), supported by artificial intelligence (AI), to measure esophageal variceal diameter (EVD) during endoscopy in previous research (9). This technology has received national utility model patent authorization. The images collected during endoscopy were re-evaluated using the objective measurement tool, effectively compensating for the subjective judgment of endoscopists. Our previous study demonstrated a correlation coefficient of 0.815 between physician visual assessments and VR measurements of EVD and an intraclass correlation coefficient of 0.965 among the measurements of three physicians using the VR, indicating consistency within and between different groups (8).

The intelligent artificial ruler can accurately measure the diameter of esophageal varices, assess bleeding risk and prognosis in PH, and guide the development of precise endoscopic and clinical treatment plans.

However, several limitations are present in this study. While clinical data were obtained from two medical centers, further validation will require data from additional institutions and larger sample sizes. Furthermore, since the clinical cases included only patients treated for bleeding events through endoscopic or interventional methods, the PPG levels were relatively high. This made it challenging to establish a relationship between EVD and PPG, particularly at lower PPG levels.

Increasing the number of cases with PPG < 20 mmHg may require the inclusion of primary prevention cases. However, conducting invasive PPG examinations may not adhere to ethical standards and could potentially harm patients, leading to increased anxiety and concern. We will continue our research and collect additional samples. It is also important to note patients with PPG < 20 mmHg have a lower risk of bleeding and a better prognosis, suggesting that those with higher PPG levels may require more focused attention.

In future studies, we will examine the relationship between EVD and the risk of rebleeding. Preliminary results indicate that patients with a diameter exceeding 1.1 cm have a higher rebleeding rate compared to those with smaller diameters. This finding can help develop more effective clinical strategies and improve patient outcomes. We recommend regular endoscopic interventions for patients with a diameter greater than 1.1 cm.

In conclusion, the findings indicate that EVD is associated with PPG levels. In patients with liver cirrhosis, an EVD greater than 1.1 cm detected endoscopically or over 1 cm observed visually may suggest a PPG exceeding 20 mmHg. Measuring EVD may provide a novel, non-invasive approach to assessing PPG and offer additional insights to aid in identifying adverse events linked to elevated PPG.

## Data Availability

The original contributions presented in the study are included in the article/supplementary material, further inquiries can be directed to the corresponding author.
